# Mutation Analysis of the *RAD51C* and *RAD51D* Genes in High-Risk Ovarian Cancer Patients and Families from the Czech Republic

**DOI:** 10.1371/journal.pone.0127711

**Published:** 2015-06-09

**Authors:** Marketa Janatova, Jana Soukupova, Jana Stribrna, Petra Kleiblova, Michal Vocka, Petra Boudova, Zdenek Kleibl, Petr Pohlreich

**Affiliations:** 1 Institute of Biochemistry and Experimental Oncology, First Faculty of Medicine, Charles University in Prague, Prague, Czech Republic; 2 Institute of Biology and Medical Genetics, First Faculty of Medicine, Charles University in Prague and General University Hospital in Prague, Prague, Czech Republic; 3 Department of Oncology, First Faculty of Medicine, Charles University in Prague, General University Hospital in Prague and Military University Hospital Prague, Prague, Czech Republic; Odense University hospital, DENMARK

## Abstract

Recent studies have conferred that the *RAD51C* and *RAD51D* genes, which code for the essential proteins involved in homologous recombination, are ovarian cancer (OC) susceptibility genes that may explain genetic risks in high-risk patients. We performed a mutation analysis in 171 high-risk *BRCA1* and *BRCA2* negative OC patients, to evaluate the frequency of hereditary *RAD51C* and *RAD51D* variants in Czech population. The analysis involved direct sequencing, high resolution melting and multiple ligation-dependent probe analysis. We identified two (1.2%) and three (1.8%) inactivating germline mutations in both respective genes, two of which (c.379_380insG, p.P127Rfs*28 in *RAD51C* and c.879delG, p.C294Vfs*16 in *RAD51D*) were novel. Interestingly, an indicative family cancer history was not present in four carriers. Moreover, the ages at the OC diagnoses in identified mutation carriers were substantially lower than those reported in previous studies (four carriers were younger than 45 years). Further, we also described rare missense variants, two in *RAD51C* and one in *RAD51D* whose clinical significance needs to be verified. Truncating mutations and rare missense variants ascertained in OC patients were not detected in 1226 control samples. Although the cumulative frequency of *RAD51C* and *RAD51D* truncating mutations in our patients was lower than that of the *BRCA1* and *BRCA2* genes, it may explain OC susceptibility in approximately 3% of high-risk OC patients. Therefore, an *RAD51C* and *RAD51D* analysis should be implemented into the comprehensive multi-gene testing for high-risk OC patients, including early-onset OC patients without a family cancer history.

## Introduction

Ovarian cancer (OC) it is the most lethal gynecologic malignancy, though it represents only 3% of female cancers worldwide. Its unfavorable prognosis related to a high percentage of late-stage diagnosis ranks OC to the fourth place among the most common cancer-related cause of death in female population [1]. Its incidence in the Czech Republic is 23/100,000 individuals and mortality amounts to 15/100,000 individuals [2].

The most important risk factor is a family OC history. It has been supposed that hereditary predisposition accounts for at least 10% of OC cases [3]. Therefore, the identification of woman carrying the pathogenic mutations in OC-susceptibility genes is an important task enabling tailored OC prevention in high-risk population. The majority of hereditary OC (HOC) cases are also associated with an increased risk of breast cancer (BC) and the most frequent pathogenic mutations in hereditary breast and/or ovarian (HBOC) cancer families affect the *BRCA1* gene and to a lesser extent also *BRCA2* [4]. Both genes play an important role in the repair of DNA double-strand breaks (DDSB). Recently, additional genes were described as associated with HOC, among them the *RAD51* paralogs *RAD51C* and *RAD51D*.

Both genes code for proteins involved in genome stability maintenance and they participate in the homologous recombination-mediated DDSB repair [5,6]. Initial studies reported the *RAD51C* gene (MIM:602774) as another Fanconi anemia (FA) gene designated as *FANCO* because its biallelic germline mutations were found in a patient with FA-like phenotype corresponding to complementation group O [7]. The pioneering work of Meindl et al. showed that *RAD51C* is an OC susceptibility gene with a mutation frequency of 1.3% in HBOC patients [8]. Since then, further studies have proved the role of the *RAD51C* gene in OC development in up to 2.5% of high-risk HBOC families [9,10,11,12] including rare large genomic rearrangements [13].

A study by Loveday et al. reported that germline mutations in the *RAD51D* gene (MIM:602954) confer susceptibility to OC in 0.9% of HBOC families (predominantly with more than one case of OC) and estimated the relative risk (RR) at 6.3 for OC and 1.32 for BC [14]. Several other studies have confirmed its role in OC susceptibility [15,16,17,18].

While individually rare, together all the moderate-penetrance genes can contribute to a significant proportion of OC risk; however, their frequencies vary in different populations [19]. The aim of our study was to determine the frequency of germline mutations in *RAD51C* and *RAD51D* among high-risk OC Czech patients negatively tested for *BRCA1* and *BRCA2* mutations.

## Methods

### Patients

We analyzed 171 DNA samples (obtained from peripheral blood) from *BRCA1* and *BRCA2* negative OC patients collected at our institute between 2000 and 2013. They belonged to either a familial group (N = 62) or a group without family cancer history (N = 109; Table 1) [20]. The control group consisted of anonymized DNA samples obtained from 1,226 individuals including 756 non-cancer individuals and 470 blood donors, as described previously [21,22].

**Table 1:** Criteria for the enrollment of high-risk *BRCA1*- and *BRCA2*-negative individuals in this study and number of identified mutations in each group.

Inclusion criteria:		Patients (N)	*RAD51C* mut (N)	*RAD51D* mut (N)
**Familial cases (N = 62)**				
	**HBOC families**:			
	- *probands with OC only*	41	1	(1)
	- *probands with both OC and BC*	9	-	-
	**HOC families** (OC ≥ 2)	12	-	-
**Cases without family cancer history (N = 109)**				
	**Tumor duplicity** (OC and BC)	22	-	-
	**OC** diagnosed **at < 60 y/o or high-grade adenocarcinoma**	87	1 (2)	3
**Total**				
		**171**	**2 (2)**	**3 (1)**

BC—breast cancer; HBOC—hereditary breast and ovarian cancer; HOC—hereditary ovarian cancer; OC—ovarian cancer; y/o—years old. Number of rare missense variants are in brackets.

All patients and controls were of Central European descent of Czech origin from the Prague region. The study was approved by the Ethical Committee of the General University Hospital in Prague and all participants gave their written informed consent with the use of stored DNA/RNA samples for research purposes.

### Mutation analysis

All individual coding exons (with intron-exon boundaries) separated by large intronic regions in the *RAD51C* gene were PCR-amplified (primers are listed in S1 Table) and analyzed by a high resolution melting (HRM) analysis on the Light Cycler 480 (Roche) using an HOT FirePol EvaGreen HRM Mix (Solis BioDyne) according to the manufacturer’s instructions.

Variants in samples with an aberrant melting profile were confirmed by direct sequencing from separate PCR reactions using the BigDye v3.1 on ABI3130 (Applied Biosystems).

All coding exons (including intron-exon boundaries) that form four exonic clusters of the *RAD51D* gene were amplified by PCR and analyzed by direct sequencing (PCR and sequencing primers are listed in S1 Table).

All exons with the identified mutations were screened in 1,226 control samples by an HRM analysis and variant samples were confirmed by sequencing.

### Detection of large genomic rearrangements (LGRs)

The P260-A1 Kit was used for a multiplex ligation-dependent probe amplification (MLPA) analysis in *RAD51C* according to the manufacturer’s protocol. The amplified products were separated on ABI3130 and analyzed by the MRC Coffalyser software (MRC Holland). The MLPA kit for *RAD51D* analysis was not available from any supplier at the time of the analysis.

### cDNA analysis

In order to determine the effect of intronic variant c.1026+5_1026+7delGTA flanking to intron-exon boundary in *RAD51C* we performed a cDNA-based analysis. The total RNA isolated from venous blood was reverse transcribed into the cDNA using SuperScript III reverse transcriptase (Life Technologies) following the manufacturer’s protocol. PCR fragment spanning the involved region was amplified (primers are in S1 Table). PCR product containing wild-type and aberrant fragments was sequenced in order to characterize aberrant splicing variant.

### *In silico* analysis

The pathogenicity of missense variants was assessed using the SIFT, PolyPhen, Align GVGD, and CADD score tools as described previously [21,22]. Frequencies of identified variants were ascertained in NHLBI Exome Sequencing Project (ESP; https://esp.gs.washington.edu/drupal/) and 1000 Genomes (http://www.1000genomes.org/) databases.

## Results

Pathogenic mutations leading to truncation of protein products were identified in five out of 171 (3%) high-risk OC patients, two (1.2%) in *RAD51C* and three (1.8%) in *RAD51D* (Table 2). Two pathogenic alterations were novel. No LGR was found in the *RAD51C* gene, but we could not exclude a presence of LGRs in the *RAD51D* gene that was not analyzed because MLPA kit was unavailable in the time of the analysis. None of the identified alterations was found in the 1,226 non-cancer controls. The age at diagnosis and the family history of cancers in the mutations’ carriers are shown in Table 2.

**Table 2:** Mutations identified in the *RAD51C* and *RAD51D* genes in analyzed high-risk patients.

Patient No.	Exon	cDNA change	Protein change	OC/other cancers; (age at diagnosis)	Histology	Grade	Stage	Cancer in family; (age at diagnosis)
***RAD51C***								
***– truncating mutations***								
218	2	c.379_380insG^**+**^	p.P127Rfs*28	OC (25)	mucinous adenocarcinoma	G3	IC	MS-OC (56), FM-BC (60)
1273	8+	c.1026+5_1026+7delGTA	p.R322Sfs*22	OC (27), Endometrial (34)	serous adenocarcinoma	low-grade		MF-Leukemia (65), FF-Gastric ca (68)
***– rare missense mutations***								
1418	4	c.641G>A^**+**^	p.R214H	OC (28), Colon ca (28)	serous cystadenoma, borderline malignancy	—	IIIC	negative
1607	7	c.947A>G^**+**^	p.H316R	OC (55), BC (69), Lung ca (72)	n.a.	n.a.	n.a.	negative
***RAD51D***								
***– truncating mutations***								
142	8	c.694C>T	p.R232*	OC (37)	serous adenocarcinoma	high-grade	IIIC	negative
2115	8	c.694C>T	p.R232*	OC (43)	serous adenocarcinoma	high-grade	IIIC	negative
1119	9	c.879delG^**+**^	p.C294Vfs*16	OC (66)	n.a.	n.a.	n.a.	D-Thyroid ca (44) & Endometrial ca (44)
***– rare missense mutation***								
107	7	c.629C>T	p.A210V	OC (33)	adenocarcinoma	n.a.	IIIA	FM-BC (n.a.), FS1-BC (56), FS2-Colon ca (47), FF-Prostate ca (72)

^**+**^—novel variants;

B—brother; BC—breast cancer; D—daughter; F—father; FF—father’s father; FM—father’s mother; FS—father’s sister; M—mother; MF—mother’s father; n.a.—not available; OC—ovarian cancer.

Note: NG_023199, NM_058216, and NP_478123 reference sequences were used for the *RAD51C* gene and reference sequences NG_031858, NM_002878, and NP_002869 were used for the *RAD51D* gene.

**Fig 1:**
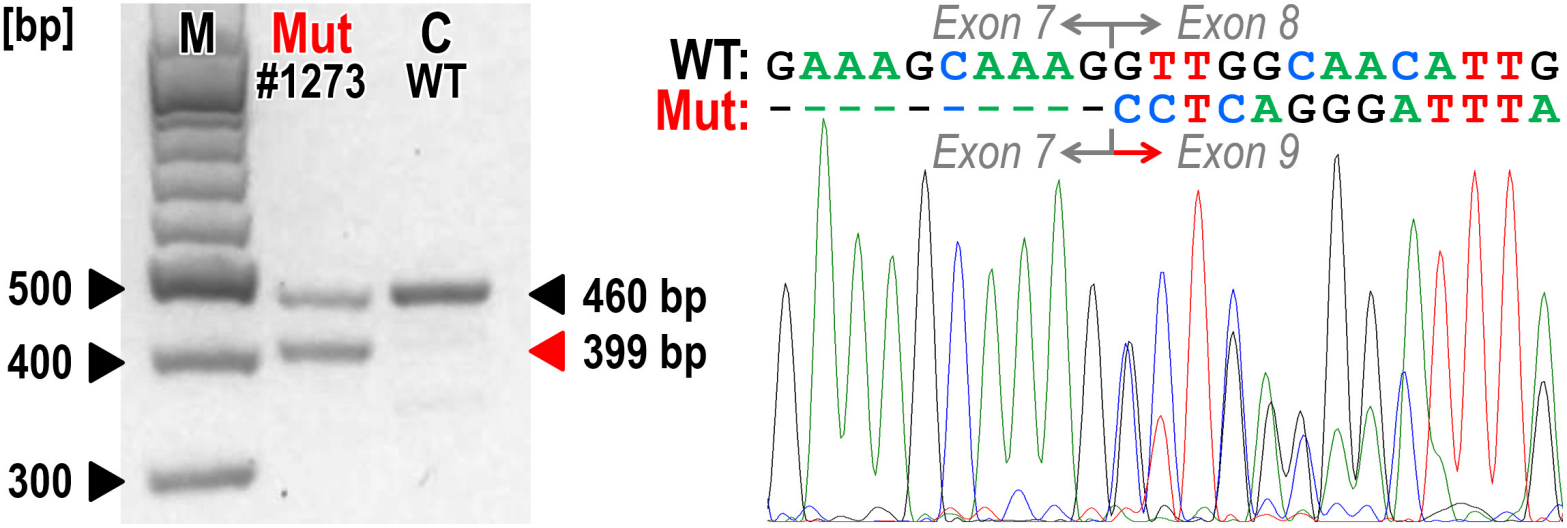
cDNA analysis of patient No.1273 uncovered with an intronic variant c.1026+5_1026+7delGTA causing RAD51C out-of-frame exon 8 skipping. The electrophoresis (left) of PCR products amplified with primers located in exon 5 and 3’ UTR sequence (S1 Table) shows two products in a patient No.1273 compared to a wild-type control (C) sample. Sequencing chromatogram of the patient’s PCR product shows the presence of aberrantly spliced mRNA with exon 8 skipping.

A novel c.379_380insG (p.P127Rfs*28) variant in *RAD51C* was found in a young OC patient who died 12 months after a diagnosis of mucinous adenocarcinoma. Another *RAD51C* truncating variant was a splice-site alteration, c.1026+5_1026+7delGTA, identified in a patient who developed endometrial carcinoma seven years after a diagnosis of ovarian serous adenocarcinoma. The pathogenic potential of this alteration was demonstrated by the cDNA analysis that revealed aberrant exon 8 skipping, that resulted in frame-shift and premature termination of translation product (p.R322Sfs*22; Fig 1). This variant was previously reported once in a control and a patient, respectively, by Loveday *et al*. and in a study by Golmard *et al*. [10,11].

The only truncating mutation described recurrently in our study was c.694C>T (p.R232*) in *RAD51D*, found in two unrelated young OC patients with serous adenocarcinoma and no family cancer history. The earlier diagnosed patient died of OC dissemination 10 years after the diagnosis. This variant was previously described in Spanish and American HBOC patients [18,16]. A novel *RAD51D* mutation, c.879delG (p.C294Vfs*16), was found in an OC patient with a daughter suffering from BC and OC duplicity.

Besides the truncating variants, we found three rare missense variants (Table 2). The c.641G>A (p.R214H) mutation in *RAD51C* was recently identified in a BC female of African origin [12] and described as a low risk variant based on her family history that is in accord with software predictions. The second rare missense variant c.947A>G (p.H316R) was novel; however, both these alterations in *RAD51C* were predicted as non-pathogenic. The only rare variant- c.629C>T (p.A210V)—in *RAD51D* was described previously [18] and was predicted as pathogenic (Table 2).

Other detected sequence variants presented in a dbSNP (http://www.ncbi.nlm.nih.gov/snp/) or HGMD (https://portal.biobase-international.com/hgmd/pro/start.php) databases as polymorphisms are listed in S3 Table.

## Discussion

We identified germline truncating mutations in the *RAD51C* and *RAD51D* genes in 1.2% and 1.8% of analyzed high-risk individuals, respectively, which is consistent with previous studies [23,24]. Our study provides further evidence that both genes confer a limited but clinically remarkable proportion of OC susceptibility.

First studies reported deleterious truncating mutations in the *RAD51C* and *RAD51D* genes predominantly in HBOC families with two or more OC cases [8,14]. However, we found the majority (four out of five clearly deleterious mutations; Table 2) in a subgroup of 109 *BRCA1* and *BRCA2* negative OC patients without BC or OC family history. This is in agreement with later studies which identified *RAD51C* and *RAD51D* mutations in an unselected population of OC patients [25,16]. These results suggest that comprehensive genetic testing of the *RAD51C* and *RAD51D* genes in OC patients should not be limited only to high-risk OC patients with an apparent family history, and an analysis of all OC patients should be considered irrespectively to the family cancer history and age of onset [26].

The mean ages of OC onset for women with *RAD51C* and *RAD51D* mutations published in previous studies were 60 years for *RAD51C* (reviewed in [23]) and 56.6 years for *RAD51D* (S4 Table). Sopik *et al*. proposed the preventive surgery to be delayed until after natural menopause. Nevertheless, in our study the mean ages of OC onset in carriers of truncating variants were 26.0 years for *RAD51C* (25 and 27 years, respectively) and 48.7 years for *RAD51D* (37, 43, and 66 years, respectively). While a small number of mutation carriers might influence this observation, we suppose that the fact that four of five carriers of pathogenic mutations in the *RAD51C* and *RAD51D* genes were premenopausal women younger than 45 years might be of clinical importance. Hence, further studies of carriers in both genes are necessary to estimate the clinical recommendations for risk-reducing bilateral salpingo-oophorectomy (RR-BSO) and until then, the earliest onset of OC in the family should be taken into account. Recently, Baker et al. reported RR-BSO in a 39 years old female BC patient carrying *RAD51D* mutation from family with multiple BC (but no OC) cases [24].

In our study, only one proband, carrying the pathogenic *RAD51C* mutation, displayed family cancer history that included OC and BC cases. Since previous studies reported no increased risk of BC for the carriers, later studies described few *RAD51C* and *RAD51D* truncating mutations in BC only families [13,27,28,18,24]. Although these mutations seem to be very rare in BC patients, they are probably responsible for certain BC predisposition. The pedigrees of mutation carriers include other cancer cases (endometrial, leukemia, thyroid), whose association with the mutations should be further analyzed in detail.

We also identified three rare missense alterations in both genes (Table 2, S2 Table). Only the c.629C>T, p.A210V rare missense variant in the *RAD51D* gene was predicted to be deleterious consistently in all used software prediction tools. However, its pathogenicity cannot be confirmed without further segregation and/or functional analyses. Unfortunately, we were not able to perform a co-segregation analysis in other family relatives.

The penetrance for mutations in moderate penetrance genes is not easy to estimate [29]. Initially, Meindl et al. reported a complete segregation of *RAD51C* mutations in HBOC families [8]. Further studies estimated the relative risk (RR) of OC for mutation carriers in *RAD51C* (RR = 5.9) and *RAD51D* (RR = 6.3) [10,14]. Pelttari *et al*. discovered an odds ratio (OR) for Finnish founder mutations in each gene in an unselected population to be 6.3 and 7.1, respectively [30,15]. These data suggest that the OC risk exceeds the threshold for moderate penetrance genes in *RAD51C* and *RAD51D* mutations carriers and both genes have clinical significance for diagnostic testing and preventive steps for the carriers involving both OC and BC management. Further studies and meta-analyses of published data from various populations worldwide are required to estimate the penetrance in *RAD51C* and *RAD51D* mutation carriers convincingly.

The clinical utility of the identification of *RAD51C* and *RAD51D* mutation carriers is not limited to the prediction of cancer susceptibility only. Both proteins are involved in DNA damage signaling and DDSB repair functionally co-operating with BRCA1 and BRCA2 in processes of homologous recombination (HR). A failure of this repair pathway that could be expected in cancer patients carrying germline pathogenic mutations in *RAD51C* and *RAD51D* sensitizes cancer cells to PARP inhibitors; therefore, patients carrying germline *RAD51C* and *RAD51D* mutations may benefit from this therapy [14].

In conclusion, we described novel pathogenic variants in *RAD51C* and *RAD51D* and showed that truncating mutations in both genes could be found in 3% of high-risk Czech OC patients. Our results indicate that the onset of OC in mutation carriers could be younger than expected. We propose that mutation analysis of *RAD51C* and *RAD51D* should be implemented into the comprehensive multi-gene panel testing in high-risk OC patients. However, further studies in various populations can help to improve the estimates of the clinical impact for germline mutation carriers and allow the determination of appropriate screening, follow-up, or prevention strategies.

## Supporting Information

S1 Table PCR primer sequences. Summary of PCR primers used for sequencing, cDNA and HRM analyses.
(DOCX)

S2 Table *In silico* prediction for missense variants. Prediction analysis of identified rare missense variants in the *RAD51C* and *RAD51D* genes and the frequency of these variants in exome sequencing and 1000 genomes projects.
(DOCX)

S3 Table *RAD51C* and *RAD51D* sequence variants. Previously described alterations (polymorphisms, intronic variants) found in our study.
(DOCX)

S4 Table Characteristics of patients carrying *RAD51D* pathogenic mutation. Ages of onset of 21 OC patients and 15 BC patients carrying *RAD51D* mutations in studies published so far.
(DOCX)
