# Supplementary material for: Mutation Analysis of the RAD51C and RAD51D Genes in High-Risk Ovarian Cancer Patients and Families from the Czech Republic
Source: PLoS One. 2015 Jun 9;10(6):e0127711. doi: 10.1371/journal.pone.0127711 (PMC4461297; doi:10.1371/journal.pone.0127711)
Supplement: S1 Table — Summary of PCR primers used for sequencing, cDNA and HRM analyses. (DOCX) [file pone.0127711.s001.docx]

**Table S1. PCR primer sequences.** Summary of PCR primers used for sequencing, cDNA and HRM analyses.

| **Primers** | **Sequence 5’ > 3’** | **Position of primers ^a/b/c^** |
| --- | --- | --- |
| ***RAD51C* ^a^** | | |
| RC_1f | TTTGGCTGCTCCGGGGTTA | 9-27 |
| RC_1r | AGGCGAGAGAACGAAGACTG | 241-260 |
| RC_2f | CTCCTAGCATCACTGTTGTCTAC | 2225-2247 |
| RC_2r | CCCACCCTTAAAAGGAGAACAC | 2599-2620 |
| RC_3f | CCTTAGATCATCATCATGATTTGG | 4043-4066 |
| RC_3r | GGTCTCAGATGGGCACAAATGC | 4308-4329 |
| RC_4f | CAATTGCCAATACATCCAAACAGG | 10539-10562 |
| RC_4r | CTTTGATTTTATGCTACTGTACTGG | 10800-10824 |
| RC_5f | CAAATCTAATATTATCTCTTCTG | 17228-17250 |
| RC_5r | CGCTATTTTGACATTTCTAGAC | 17450-17471 |
| RC_6f | AGACTGGTCTACTTGATAATTTTC | 28081-28104 |
| RC_6r | CTCAATGAGAATCAAATGAAAGAG | 28261-28284 |
| RC_7f | ACCAAGTCAGTAAGGCCATATAC | 31392-31414 |
| RC_7r | CACAGGACTAGCTCTAAGAAACC | 31564-32586 |
| RC_8f | CTCTCCTTTTTGTGTTCTTAGAG | 39742-39764 |
| RC_8r | CCCATCAATATCAAAATCCCACTC | 39962-39985 |
| RC_9f | GATCAGTCTTCAAATGTTCTTAAAGC | 41461-41486 |
| RC_9r | TAACAAGTCCACTTGTACACATTG | 41669-41692 |
| ***RAD51C* ^b^ for cDNA analysis** | | |
| R_RC_5F | CGTCATGACCTAGATGACCTG | c.745-765 |
| R_RC_9r | GGTAACAAGTCCACTTGTACAC | c.1131+52-1131+73 |
| ***RAD51D* ^c^** | | |
| RD_1f* | GCCTCCTCCTCTCTCCTTTC | 110-129 |
| RD_2f | GGTAGAATTGACACCCCATTTG | 603-624 |
| RD_2r | TGACTTCTGACTCCAAGTGACC | 979-1000 |
| RD_3r* | TGTCCTGACCCCTTTCCTTC | 1489-1508 |
| RD_4f* | TGGCCAGTGATGTTCAAAGA | 12300-12319 |
| RD_5f | GACTCAGCCCATTTGTGTTG | 12644-12663 |
| RD_5r | AGCAAGTTTGAAGGCAAGGA | 12974-12993 |
| RD_6r* | GATTGCACATCTGCATTTCCA | 13569-13589 |
| RD_7f* | CAGCTTGCTGTATTTGGGATG | 16203-16223 |
| RD_8r* | TTTGGGGTTCAGAAGCTGAC | 16684-16704 |
| RD_9f* | TCTCCGTAAAATGAAGCGGTAG | 18428-18449 |
| RD_10r* | TAAACAGCAGGCGTTACTGG | 18975-18994 |

Note: NCBI reference sequences: ^a^ – NG_023199 used for *RAD51C* gene; ^b^ – NM_058216 used for RAD51C mRNA; ^c^ – NG_031858 used for *RAD51D* gene. (*) denotes primers used for amplification of four genomic regions containing clustered coding exons 1-3, 4-6, 7-8, and 9-10.
