# Supplementary material for: Mutation Analysis of the RAD51C and RAD51D Genes in High-Risk Ovarian Cancer Patients and Families from the Czech Republic
Source: PLoS One. 2015 Jun 9;10(6):e0127711. doi: 10.1371/journal.pone.0127711 (PMC4461297; doi:10.1371/journal.pone.0127711)
Supplement: S2 Table — Prediction analysis of identified rare missense variants in the RAD51C and RAD51D genes and the frequency of these variants in exome sequencing and 1000 genomes projects. (DOCX) [file pone.0127711.s002.docx]

**Table S2. *In silico* prediction for missense variants.** Prediction analysis of identified rare missense variants in the *RAD51C* and *RAD51D* genes and the frequency of these variants in exome sequencing and 1000 genomes projects.

| **Gene** | **Protein change** | **GVGD** | **Polyphen** | **SIFT** | **CADD score** | **ESP** | **1000 genomes** |
| --- | --- | --- | --- | --- | --- | --- | --- |
| ***RAD51C*** | p.R214H^+^ | C0 | Benign (0.001) | Tolerated (0.39) | 15.16 | -- | -- |
|  | p.H316R^+^ | C25 | Benign (0.002) | Tolerated (1.00) | 20.8 | -- | -- |
| ***RAD51D*** | p.A210V | C65 | Damaging (1.000) | Damaging (0.01) | 33 | -- | -- |

^+^ – novel variants.
