# Supplementary material for: Mutation Analysis of the RAD51C and RAD51D Genes in High-Risk Ovarian Cancer Patients and Families from the Czech Republic
Source: PLoS One. 2015 Jun 9;10(6):e0127711. doi: 10.1371/journal.pone.0127711 (PMC4461297; doi:10.1371/journal.pone.0127711)
Supplement: S3 Table — Previously described alterations (polymorphisms, intronic variants) found in our study. (DOCX) [file pone.0127711.s003.docx]

**Table S3.** ***RAD51C* and *RAD51D* sequence variants.** Previously described alterations (extracted from all relevant studies published so far) that was also identified in our study.

| **Gene** | **Exon** | **Variant** | **Protein change** | **rs number** | **No of cases** | **CADD score** | **ESP** | **1000 genomes** |
| --- | --- | --- | --- | --- | --- | --- | --- | --- |
| ***RAD51C*** | 2 | c.376G>A | p.A126T | [rs61758784](http://www.ncbi.nlm.nih.gov/projects/SNP/snp_ref.cgi?rs=61758784) | 2 | 21.4 | 0.004613 | 0.0009 |
|  | 4- | c.572-17G>T | -- | rs193023469 | 1 | 14.72 | 0.005999 | 0.0032 |
|  | 6 | c.859A>G | p.T287A | [rs28363317](http://www.ncbi.nlm.nih.gov/projects/SNP/snp_ref.cgi?rs=28363317) | 1 | 26.1 | 0.007843 | 0.01 |
| ***RAD51D*** | 1+ | c.82+128C>T | -- | rs28363258 | 4 | 10.24 | -- | 0.0046 |
|  | 2 | c.141C>T | p.(=) | -- | 1 | 20.9 | -- | -- |
|  | 3 | c.234C>T | p.(=) | [rs9901455](http://www.ncbi.nlm.nih.gov/projects/SNP/snp_ref.cgi?rs=9901455) | 43 | 22.2 | 0.158542 | 0.19 |
|  | 3+ | c.263+63_263+65delGCT | -- | -- | 1 | -- | -- | -- |
|  | 5- | c.481-8C>T | -- | -- | 1 | 9.098 | -- | -- |
|  | 6 | c.494G>A | p.R165Q | rs4796033 | 66 | 17.54 | 0.106797 | 0.1 |
|  | 8- | c.668-36C>T | -- | -- | 1 | 3.774 | -- | -- |
|  | 8 | c.698A>G | p.E233G | [rs28363284](http://www.ncbi.nlm.nih.gov/projects/SNP/snp_ref.cgi?rs=28363284) | 4 | 25 | 0.012686 | 0.01 |
|  | 9+ | c.903+53C>T | -- | rs45496096 | 8 | 6.281 | -- | 0.01 |

Note: NCBI reference sequences: NM_058216 used for *RAD51C* mRNA; NM_002878 used for *RAD51D* mRNA.
