# Supplementary material for: Mutation Analysis of the RAD51C and RAD51D Genes in High-Risk Ovarian Cancer Patients and Families from the Czech Republic
Source: PLoS One. 2015 Jun 9;10(6):e0127711. doi: 10.1371/journal.pone.0127711 (PMC4461297; doi:10.1371/journal.pone.0127711)
Supplement: S4 Table — Ages of onset of 21 OC patients and 15 BC patients carrying RAD51D mutations in studies published so far. (DOCX) [file pone.0127711.s004.docx]

**Table S4.** **Characteristics of patients carrying *RAD51D* pathogenic mutation.** Ages of onset of 21 OC patients and 15 BC patients carrying *RAD51D* mutations in studies published so far.

| Study | Patient  OC | Patient  BC | Mutation | Cancer diagnosis | Age  of onset | Proband status |
| --- | --- | --- | --- | --- | --- | --- |
| Loveday *et al*.[1] | - | 1 | c.363delA | BC left | 34,52* | proband |
|  | 1 | - | c.803G>A | OC | 58 | proband |
|  | 1 | - | c.556C>T | OC | 38 | proband |
|  | - | 1 |  | BC | 39 | relative |
|  | - | 1 |  | BC | 58 | relative |
|  | - | 1 |  | BC | 53 | relative |
|  | - | 1 | c.480+1G>A | BC | 51 | proband |
|  | 1 | - | c.345G>C | OC | 45 | proband |
|  | 1 | - |  | OC | 74 | relative |
|  | - | 1 | c.556C>T | BC | 35 | proband |
|  | 1 | - | c.757C>T | OC | 51 | proband |
|  | - | 1 |  | BC | 47 | relative |
|  | 1  - | -  1 | c.270_271dupTA | OC | 58 | proband |
|  |  |  |  | BC | 65 |  |
| Osher *et al.*[2] | - | 1 | c.556C>T | DCIS | 47 | proband |
|  | - | 1 |  | BC | 34 | relative |
|  | 1 | - |  | OC | 56 | relative |
|  | 1 | - |  | OC | 59 | relative |
| Pelttari *et al.*[3] | - | 2 | c.576+1G>A | BC mean | 45.7  (35.7-61.4) |  |
|  | 5 | - |  | OC mean | 66.5  (53.6-79) |  |
| Wickramanyake *et al.*[4] | 1 | - | c.580delA | OC | 33 | proband |
|  | 1 | - | c.694C>T | OC | 43 | proband |
|  | 1 | - | c.131_134+24del38 | OC | 75 | proband |
| Thompson *et al.*[5] | 1 | - | c.556C>T | OC | 66 | proband |
|  | 1 | - | c.803G>A | OC | 70 | proband |
| Gutiérrez-Enríquez *et al.*[6] | 1 | - | c.1A>T | OC | 44 | proband |
|  | - | 1 |  | BC | 51 |  |
|  | - | 1 | c.667+2_667+23del | BC | 29,40* | proband |
|  | - | 1 |  | BC | 49 | relative |
|  | - | 1 |  | BC | 43,43* | relative |
|  | 1 | - | c.694C>T | OC | 44 | proband |
|  | 1 | - | c.694C>T | OC | 42 | proband |
| **Total mean age - OC** | 21 | - |  | **OC** | **54.3 (56.6)** |  |
| **- BC** | - | 15 |  | **BC** | **45.4 (45.4)** |  |

* - the only earlier onset was considered in case of patients with bilateral BC.

**Supporting references**

1. Loveday C, Turnbull C, Ramsay E, Hughes D, Ruark E et al. (2011) Germline mutations in RAD51D confer susceptibility to ovarian cancer. Nat Genet 43: 879-882.

2. Osher DJ, De Leeneer K, Michils G, Hamel N, Tomiak E et al. (2012) Mutation analysis of RAD51D in non-BRCA1/2 ovarian and breast cancer families. Br J Cancer 106: 1460-1463.

3. Pelttari LM, Kiiski J, Nurminen R, Kallioniemi A, Schleutker J et al. (2012) A Finnish founder mutation in RAD51D: analysis in breast, ovarian, prostate, and colorectal cancer. J Med Genet 49: 429-432.

4. Wickramanayake A, Bernier G, Pennil C, Casadei S, Agnew KJ et al. (2012) Loss of function germline mutations in RAD51D in women with ovarian carcinoma. Gynecol Oncol 127: 552-555.

5. Thompson ER, Rowley SM, Sawyer S, kConfab, Eccles DM et al. (2013) Analysis of RAD51D in ovarian cancer patients and families with a history of ovarian or breast cancer. PLoS One 8: e54772.

6. Gutierrez-Enriquez S, Bonache S, de Garibay GR, Osorio A, Santamarina M et al. (2014) About 1% of the breast and ovarian Spanish families testing negative for BRCA1 and BRCA2 are carriers of RAD51D pathogenic variants. Int J Cancer 134: 2088-2097.
